# Pressurized intraperitoneal aerosol chemotherapy (PIPAC): updated systematic review using the IDEAL framework

**DOI:** 10.1093/bjs/znac284

**Published:** 2022-09-03

**Authors:** Alice E Baggaley, Guillaume B R C Lafaurie, Sophia J Tate, Piers R Boshier, Amy Case, Susan Prosser, Jared Torkington, Sadie E F Jones, Sarah H Gwynne, Christopher J Peters

**Affiliations:** Department of Surgery and Cancer, Imperial College London, St Mary’s Hospital, London, UK; Department of Surgery and Cancer, Imperial College London, St Mary’s Hospital, London, UK; Department of Anaesthesia, Swansea Bay University Health Board, Swansea, UK; Department of Surgery and Cancer, Imperial College London, St Mary’s Hospital, London, UK; Department of Cancer Services, Swansea Bay University Health Board, Swansea, UK; Department of Library Services, Swansea Bay University Health Board, Swansea, UK; Department of Surgery, University Hospital of Wales, Cardiff, UK; Department of Obstetrics and Gynaecology, University Hospital of Wales, Cardiff, UK; Department of Cancer Services, Swansea Bay University Health Board, Swansea, UK; Department of Surgery and Cancer, Imperial College London, St Mary’s Hospital, London, UK

## Introduction

Pressurized intraperitoneal aerosol chemotherapy (PIPAC) is a surgical innovation deployed to treat peritoneal metastases. Traditionally, peritoneal metastases have been treated with systemic chemotherapy, but this approach is limited by poor peritoneal perfusion. Intra-abdominal chemotherapy in the form of heated lavage (hyperthermic intraperitoneal chemotherapy (HIPEC)) is currently used alongside cytoreductive surgery. The use of aerosolized agents in a laparoscopic setting was first described in 2000 in a swine model^1^; since then, a number of PIPAC studies have been reported. The IDEAL framework^2^ provides recommendations for the design, development, and reporting of studies for novel surgical interventions (*Table 1*). It recommends that innovations move through stages (idea, development, exploration, assessment, and long-term studies).

**Table 1:** Summary of the stages of surgical innovation according to the IDEAL framework

Stage of innovation	Description	No. of patients	Proposed method of investigation	Studies included in this review
**0: idea (preclinical)**	Feasibility and definition of procedure	None	Simulated, cadaver, animal, modelling	Preclinical studies in animals (*in vivo* and post-mortem models) and *in vitro*
**1: idea**	Proof of concept; first in human	Very few	Case reports, small case series	Case reports, small case series, occupational health and safety studies; data relate to safety and technical feasibility
**2a: development**	Therapy evolving; refining and modifying the technique	Usually < 30	Prospective development studies	Larger case series, non-randomized studies; prospective and retrospective case series; single centre
**2b: exploration**	Learning curves progressing, indication expanding	Many	Prospective series, multisite, feasibility RCT	Large multicentre case series, studies looking at new indication
**3: assessment**	Procedure has clear definition and used by many surgeons but needs to be tested against standard of care	Many	RCT	RCT and RCT protocols
**4: long-term**	Long-term follow-up with registry data, to monitor late/rare complications	Many	Registry, late/rare case reports	NA

RCT, randomized clinical trial; NA, not applicable.

This paper provides an update of the previously performed PIPAC IDEAL review^3^, to include updated research. There are almost double the number of PIPAC papers now (165 *versus* 86), compared with the search completed 3 years ago. This review was performed on behalf of the PIPAC UK collaborative.

## Methods

This systematic review was conducted with the MEDLINE and Embase databases, up to 28 February 2022. Included studies were assigned a stage (0, 1, 2a, 2b, 3, or 4), using the IDEAL guidelines^4^. Full methodological details, including the PRISMA checklist/flow chart, are available in the *Supplementary material*.

## Results

After screening, 18 trial registrations and 147 published papers were included^1,5–151^. IDEAL stage allocation can be viewed online (*Supplementary material*).

### Stage 0: idea (preclinical)

The first description of a ‘therapeutic pneumoperitoneum’ in a swine model was published in 2000^1^. Studies successfully demonstrated the superiority of PIPAC over conventional lavage with regard to peritoneal distribution and drug penetration using methylene blue dye, and Dbait with a fluorescent marker^5,6^. Further studies demonstrated drug penetration was highest closest to the delivery device, and that aerosol distribution was heterogenous^7–9^. Studies found that increasing the intra-abdominal pressure to 15 mmHg (from 12 mmHg) increased the cytotoxic action of oxaliplatin on a cell line^10^, but a higher temperature did not have a significant effect.

Some units investigated how to improve chemotherapy delivery; demonstrating the stability of nano- or microparticles during PIPAC^11,12^. Further experiments addressed non-homogenous drug distribution with the use of a rotational/multidirectional nozzle^13,14^. Another modification involved the use of electrostatic precipitation, and was named ePIPAC^15^.

### Stage 1: idea

The first in-human studies performed on patients with peritoneal metastases were published in 2013 and 2014^16,17^. They demonstrated peritoneal tumour regression in the three patients treated, with limited renal and liver toxicity. The PIPAC technique was described as follows: pressurized aerosolization of cisplatin and doxorubicin; 12 mmHg CO_2_ pneumoperitoneum over 30 minutes; and a temperature of 37°C. The dosage of cisplatin (7.5 mg/m^2^ body surface) and doxorubicin (1.5 mg/m^2^ body surface) were set as 10 per cent of the usual HIPEC dose. Occupational health studies demonstrated safety for theatre staff^18^, with most new PIPAC groups each performing their own occupational safety tests^19–25^.

### Stage 2a: development

Early perioperative complications included rare but life-threatening instances of severe peritoneal sclerosis or severe hypersensitivity reactions to platinum^26,27^. A systematic review included 28 clinical studies involving more than 1500 patients, and showed that 45 per cent of patients developed a grade 1 adverse event, but only 1.6 per cent of patients had a grade 5 adverse event (death)^42^. No significant renal, hepatic, or haematological toxicity was described. One study assessed quality of life (QoL) in 91 patients and demonstrated no therapy-related decrease in QoL score^28^.

The most common PIPAC chemotherapy regimen is either oxaliplatin as a sole agent or cisplatin with doxorubicin. Formal dose-escalation studies include a phase 1 study that found patients undergoing PIPAC could tolerate an increase in the dose of cisplatin and doxorubicin up to 10.5 and 2.1 mg/m^2^, respectively^29^. Another unit found that the maximum tolerated dose of oxaliplatin was 90 mg/m^2^^30^. However, another phase 1 dose-escalation study found that three patients could tolerate a maximum dose of 135 mg/m^2^^31^, with no dose-limiting toxicity observed. They also looked at cisplatin and doxorubicin, and found a maximum tolerated dose of 30 mg/m^2^ and 6 mg/m^2^, respectively—significantly higher than doses used in any previous PIPAC application. Common adverse events across all these studies included nausea, vomiting, and abdominal pain. While earlier trials assessed PIPAC in ovarian and colorectal peritoneal metastases, indications have expanded, and include cholangiocarcinoma^32^, pancreas^33^, breast and endometrial origins^35^.

### Stage 2b: exploration

There has been rapid expansion of PIPAC from Germany^28,35^ to nearby countries, including France, Switzerland, and the Netherlands^36–38^. Its wide acceptance into practice has led some papers to describe as many as 1200 PIPAC treatments^49^. Its safety has been demonstrated, with minimal risks and impact on QoL, and ePIPAC has been shown to be feasible, safe, and repeatable in patients^40^. There is also evidence that PIPAC may be used as a neoadjuvant treatment, with downstaging of peritoneal disease enabling transition from unresectable to resectable tumours in a small number of patients^41^.

### Stage 3: assessment

The penultimate IDEAL stage involves testing the proposed surgical innovation against the standard of care. To date there have been six stage 3 published protocols, but no results have yet been published. Half of the protocols compare cycles of PIPAC + systemic chemotherapy with systemic chemotherapy; the other half compare PIPAC alone with systemic chemotherapy. Both the disease targeted and the primary outcomes evaluated are variable. The lack of a consistent outcome measurement in these trials may make it more difficult to compare results. Two-thirds of the proposed studies will be multicentred, with collaboration across the European PIPAC units.

## Discussion

**Fig. 1:**
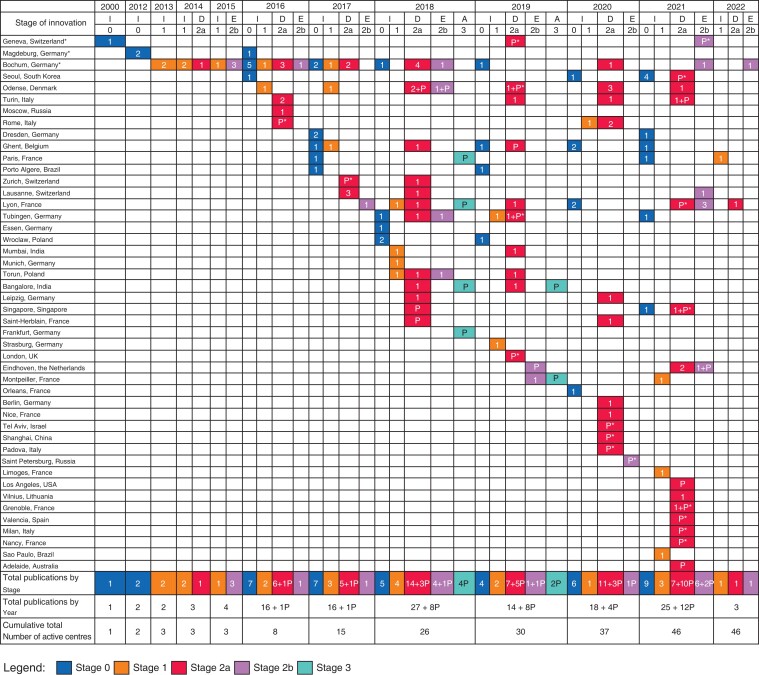
Identified studies were assigned a stage and displayed by year and location Study centres are described by city where the institution of the lead author was located, or where the pressurized intraperitoneal aerosol chemotherapy (PIPAC) was performed. An asterisk (*) next to location indicates the original PIPAC group moved (now Bochum). I, idea; D, development; E, exploration; A, assessment; *P*, published protocol paper; P*, protocol from ClinicalTrials.gov or clinicaltrialsregister.eu.

Since 2019, there has been an increase in the number of studies on PIPAC published (165 *versus* 86), as well as the number of units using PIPAC (46 *versus* 28). As *Fig. 1* demonstrates, there is a general progression through the IDEAL stages, although published randomized clinical trials (RCTs) are still lacking. A PIPAC online registry (https://isspp.org) has been set up by the International Society of the Study of Pleura and Peritoneum and, if utilized by the PIPAC community, should provide the foundation for future stage 4 reports.

According to the IDEAL framework, surgical innovation should progress through the stages in a step-wise fashion, but this does not mean that each new unit need regress to stage 0 if little is being changed. This paper also highlights the need for all clinical trials to be prospectively registered, as only a fraction appear prospectively on clinical trials registries, if at all.

Given that the use of PIPAC to treat peritoneal disease has been practised, mostly in Europe, for the past decade, it is imperative that robust RCTs are set up to compare this intervention with the standard of care. There is a risk that in some units the use of PIPAC is so widespread that it may be a barrier to patient recruitment into the non-PIPAC arm within a RCT. The lack of robust evidence for efficacy means that in the UK PIPAC remains categorized within the National Institute of Health and Care Excellence guidelines for use in clinical trials only^152^. The PIPAC UK collaborative has been formed in response to this recommendation. Through the collaboration, the UK is ideally placed to carry out a multicentre RCT. This would allow the effectiveness of PIPAC to be demonstrated definitively and place this innovation within routine care pathways.

## Collaborators

Amy Case: Swansea Bay University Health Board. Swansea, Wales. Angela Casbard: Cardiff University. Cardiff, Wales. Chris Peters: Imperial College London. London, England. David Chuter: Royal Surrey County Hospital. Guildford, England. Emma Hudson: Velindre University NHS Trust. Cardiff, Wales. Gina Brown: Imperial College London. London, England. Harry Hall: Imperial College Healthcare NHS Trust. London, England. Jamie Murphy: Imperial College London. London, England. Jared Torkington: Cardiff and Vale University Health Board. Cardiff, Wales. Jody Parker: Cardiff and Vale University Health Board. Cardiff, Wales. Jonathan Frost: Royal United Hospitals Bath NHS Foundation Trust, Bath, England Joy Garfitt: Cardiff and Vale University Health Board. Cardiff, Wales. Kitrick Perry: Imperial College Healthcare NHS Trust, London, England Leona Batten: Cardiff University. Cardiff, Wales. Lisette Nixon: Cardiff University. Cardiff, Wales. Peter Kyle: Imperial College London. London, England. Richard Adams: Cardiff University. Cardiff, Wales. Sarah Gwynne: Swansea Bay University Health Board. Swansea, Wales. Sadie Jones: University Hospital of Wales. Cardiff, Wales. Sophie Tate: Swansea Bay University Health Board. Swansea, Wales. Steve Kihara: Swansea Bay University Health Board. Swansea, Wales. Alan Parker: Cardiff University. Cardiff, Wales. Alice Baggaley: Imperial College London. London, England.

## Supplementary Material

znac284_Supplementary_Data

## Data Availability

Data obtained from the IDEAL review process is available on request.
