# Supplementary material for: Pressurized intraperitoneal aerosol chemotherapy (PIPAC): updated systematic review using the IDEAL framework
Source: Br J Surg. 2022 Sep 3;110(1):10–8. doi: 10.1093/bjs/znac284 (PMC10364525; doi:10.1093/bjs/znac284)
Supplement: znac284_Supplementary_Data [file znac284_supplementary_data.zip › Supplementary_material.docx]

**Supplementary material**

**Methods**

A systematic search was conducted on Medline and Ovid databases using search terms to include studies involving PIPAC and peritoneal metastases, irrespective of cancer origin, up to 28th February 2022 (three years from original PIPAC IDEAL review). PIPAC *in vitro* and animal studies, occupational health and technology studies, and case reports were all included in this review. Conference abstracts, chapters, reviews, and papers not published in English were excluded. ClinicalTrials.gov and the EU Clinical Trials register were searched for trial registrations involving PIPAC. Included studies were categorised into IDEAL stages and sorted by year published and location of the research group.

**PRISMA flowchart**


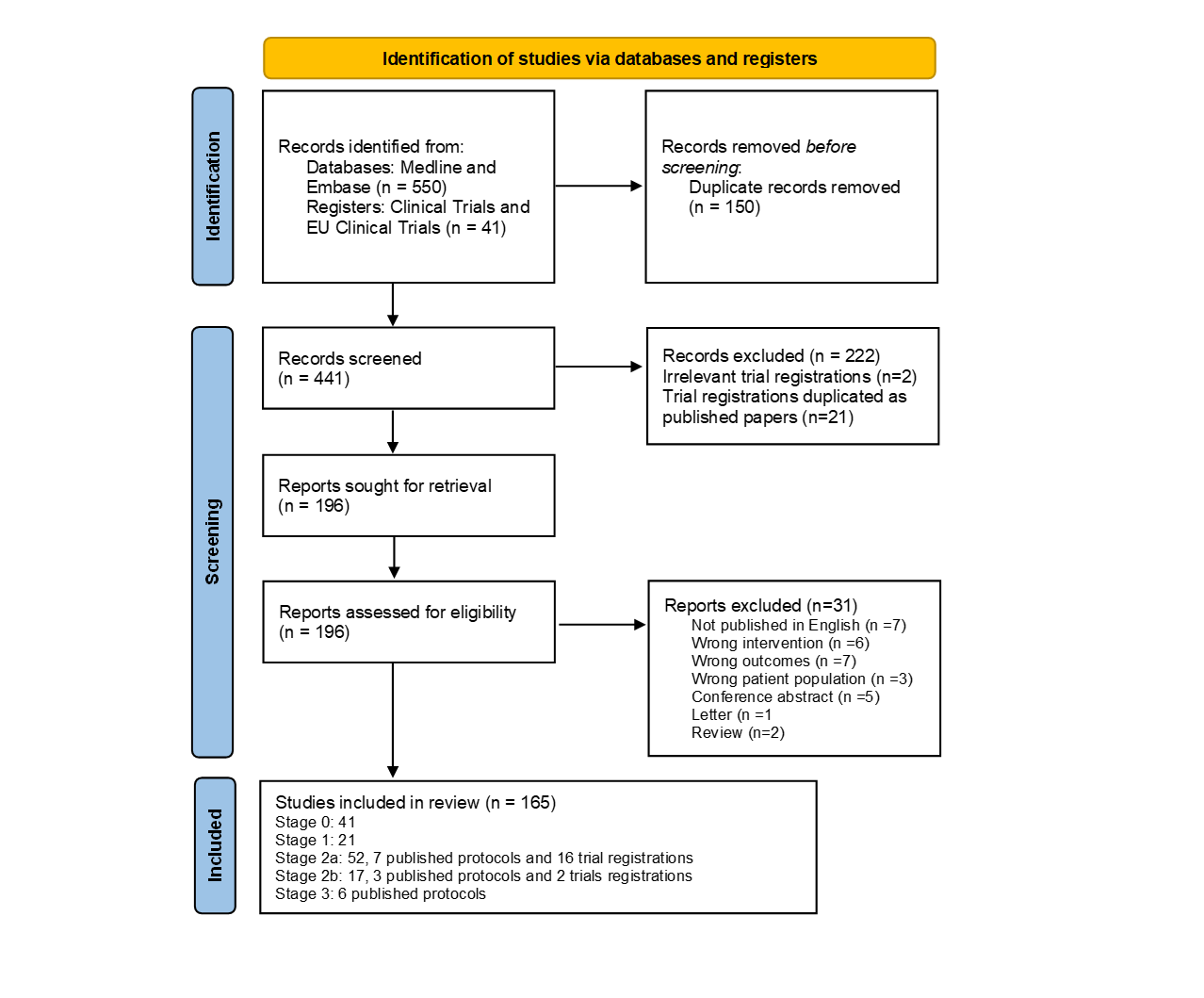


**Search terms**

**Ovid MEDLINE(R) ALL <1946 to February 28th, 2022>**

1 ((pressur* or laparoscopic*) adj4 (intra-periton* or intra?periton* or "intra periton*" or intra-abdominal* or intra?abdominal or "intra abdominal*") adj4 (chemo?therap* or chemo or therap* or treat*)).tw.

2 (electrostatic* adj4 pressur* adj4 (intra-periton* or intra?periton* or "intra periton*" or intra-abdominal* or intra?abdominal or "intra abdominal*") adj4 (chemo?therap* or chemo or therap* or treat*)).tw.

3 PIPAC*.tw.

4 (ePIPAC* or PITAC*).tw.

5 (carcinomato* or carcino* or metast* or neoplas* or cancer or malign* or tumo?r* or lump*).tw.

6 exp Neoplasms/

7 5 or 6

8 1 or 2 or 3 or 4

9 7 and 8

**OVID Embase <1996 to 2022 February 28th>**

1 ((pressur* or laparoscopic*) adj4 (intra-periton* or intra?periton* or "intra periton*" or intra-abdominal* or intra?abdominal or "intra abdominal*") adj4 (chemo?therap* or chemo or therap* or treat*)).tw.

2 (electrostatic* adj4 pressur* adj4 (intra-periton* or intra?periton* or "intra periton*" or intra-abdominal* or intra?abdominal or "intra abdominal*") adj4 (chemo?therap* or chemo or therap* or treat*)).tw.

3 PIPAC*.tw.

4 (ePIPAC* or PITAC*).tw.

5 (carcinomato* or carcino* or metast* or neoplas* or cancer or malign* or tumo?r* or lump*).tw.

6 exp neoplasm/

7 5 or 6

8 1 or 2 or 3 or 4

9 7 and 8
